# Cellular Mutagenicity and Heavy Metal Concentrations of Leachates Extracted from the Fly and Bottom Ash Derived from Municipal Solid Waste Incineration

**DOI:** 10.3390/ijerph13111078

**Published:** 2016-11-02

**Authors:** Po-Wen Chen, Zhen-Shu Liu, Min-Jie Wun, Tai-Chen Kuo

**Affiliations:** 1 Department of Nursing, St. Mary’s Junior College of Medicine, Nursing and Management, Yilan 26644, Taiwan; pwchen@smc.edu.tw; 2 Department of Safety, Health and Environmental Engineering, Ming Chi University of Technology, New Taipei City 24301, Taiwan; dream_5788@yahoo.com.tw (M.-J.W.); aa701623@yahoo.com.tw (T.-C.K.)

**Keywords:** fly ash, bottom ash, mutagenicity, Ames test, toxicity characteristic leaching procedure (TCLP)

## Abstract

Two incinerators in Taiwan have recently attempted to reuse the fly and bottom ash that they produce, but the mutagenicity of these types of ash has not yet been assessed. Therefore, we evaluated the mutagenicity of the ash with the Ames mutagenicity assay using the TA98, TA100, and TA1535 bacterial strains. We obtained three leachates from three leachants of varying pH values using the toxicity characteristic leaching procedure test recommended by the Taiwan Environmental Protection Agency (Taiwan EPA). We then performed the Ames assay on the harvested leachates. To evaluate the possible relationship between the presence of heavy metals and mutagenicity, the concentrations of five heavy metals (Cd, Cr, Cu, Pb, and Zn) in the leachates were also determined. The concentrations of Cd and Cr in the most acidic leachate from the precipitator fly ash and the Cd concentration in the most acidic leachate from the boiler fly ash exceeded the recommended limits. Notably, none of the nine leachates extracted from the boiler, precipitator, or bottom ashes displayed mutagenic activity. This data partially affirms the safety of the fly and bottom ash produced by certain incinerators. Therefore, the biotoxicity of leachates from recycled ash should be routinely monitored before reusing the ash.

## 1. Introduction

The municipal solid waste (MSW) resource recovery program has been promoted in Taiwan since 1998. According to the latest data from the Taiwan Environmental Protection Agency (TEPA), approximately 7.4 million metric tons (MT) of MSW were produced in 2014. The total resource recovery rate reached 55.78% in 2014 [1]. After recovery, surplus MSW mainly undergoes incineration. At present, there are 26 total (24 functional) incinerators in Taiwan, which processed approximately 6.3 million MT of general and industrial waste in 2014. This ultimately led to the production of approximately 0.937 million MT (14.89%) of bottom ash (BA) and 0.194 million MT (3.09%) of fly ash (FA) [2]. 

Because BA is often considered nonhazardous, it can be reused for road construction and cement manufacturing [3,4,5]. In 2014, 77.38% of the BA produced in Taiwan was recycled [2]. However, recycled ash may still contain heavy metals in relatively high concentrations and organic compounds in low concentrations [6]. Several studies have provided information regarding the leaching of heavy metals from BA [5,6,7,8]. By contrast, FA is often considered hazardous [5] because of its generally higher concentrations of heavy metals. Therefore, most FA is instead solidified and placed in landfills. In 2014, the solidification of FA produced approximately 0.29 million MT of solidified ash in Taiwan.

Recycling FA may be a better alternative to solidification. Two incinerators in Taiwan have recently started to recycle a portion of their FA. In 2014, one of these incinerators recycled 100% of its FA (approximately 3134 MT), and the other recycled nearly 10% (661 MT). Together, these figures represent only 1.31% (3795 MT) of the total FA produced in Taiwan [2]. Before FA is reused, the chloride and a portion of the heavy metals are removed by water washing; the processed ash is then used for cement manufacturing. One reason for this relatively low recycling rate is that the potential environmental impacts of FA are not yet fully understood. As more FA is recycled, assessing the chemical composition of the derived leachates and the risk of biotoxicity becomes increasingly crucial. To evaluate these attributes, we tested samples from a Taiwanese incinerator that recycles a high proportion of its FA using the Ames assay.

Several approaches have been used to determine the ecotoxicological impact of MSW incineration (MSWI) ash [9,10,11,12,13,14] and other solid waste [15,16,17,18,19,20,21]. The Ames Salmonella mutagenicity assay has been widely used to determine the carcinogenic potential of a variety of substances [15,22,23,24,25,26,27,28]. In a recent study [8], we applied this assay to determine the mutagenic activity of BA collected from an incinerator in Taiwan. Our data indicated that while the heavy metal contents in leachates were below the Taiwanese regulatory limits, certain leachates from both non-sieved and <4.75 mm-sieved BA showed mutagenicity. From these findings, we concluded that the chemical composition and mutagenic potential of leachates should be analyzed simultaneously.

To the best of our knowledge, this is the first study to apply the Ames test to FA samples from Taiwan. Notably, the pH values of leachates A (pH 4.93), B (pH 2.88), and C (deionized water, pH 6.0) and the extreme alkaline solutions did not contribute to cellular cytotoxicity when the Ames/Salmonella mutagenicity test was used to evaluate the mutagenicity of incineration ash leachate, as shown in our previous study [8]. In the previous study, a Vibrio fischeri light inhibition test was used to evaluate the biotoxicity of FA and BA leachates from different MSW incinerators. The results indicated that pH influenced the biotoxicity of the leachate, and the extremely alkaline solutions (pH 11.7) were toxic to V. fischeri [13]. Therefore, the Ames/Salmonella mutagenicity test is more appropriate for evaluating the biotoxicity of incineration ash leachate. The Ames assay has previously been applied to FA from MSWI [29,30,31]. For instance, the Ames assay has been applied to FA from 16 municipal waste incineration plants in Japan [31], and the results indicated that FA from six of the incinerators displayed mutagenicity. However, boiler fly ash (BFA) and precipitator fly ash (PFA) were not analyzed individually in previous studies. Previous studies support that the Ames assay should be routinely applied to monitor the mutagenic potential of both FA and BA to determine the possible environmental impacts.

FA and BA leachates were obtained using the toxicity characteristic leaching procedure (TCLP), as recommended by the Taiwan EPA. Three leachants of varying pH values were used for the TCLP: leachant A (pH 4.93), leachant B (pH 2.88), and leachant C (deionized water, pH 6.0). Inductively coupled plasma-optical emission spectrometry (ICP-OES) was used to determine the concentrations of five key heavy metals (Cd, Cr, Cu, Pb, and Zn) in all nine leachates. Then, the Ames assay was performed on the leachates.

## 2. Materials and Methods

### 2.1. Collection of Fly Ash and Bottom Ash

Three types of MSWI ash—BA, BFA, and PFA—were collected for this study, following the method for sampling of waste incineration ash (NIEA R119.00C) recommended by the Taiwan EPA. All three types of ashes were obtained from the same incinerator in Taiwan, which has been in use since 1995 and has an operational capacity of 1200 tons per day. The furnace is a mechanical-grate incinerator, and its temperature ranges from 850 to 1050 °C. Four sets of boilers are used to recover waste heat from the incinerator. The air pollution control device (APCD) is an electrostatic precipitator (ESP) integrated with a wet scrubber and a catalytic reactor. The BA, BFA, and PFA were collected from the incinerator, the boiler, and the ESP, respectively. In this study, the BA, BFA, and PFA were dried in an oven at 105 °C for 1 h and were then subjected to the leaching procedure. 

### 2.2. Leaching Procedure

The leaching procedure essentially followed the TCLP test (NIEA R201.14C) procedure described by the Taiwan EPA, except that deionized water and acetic acid (CH_3_COOH) were used as the leaching agents [8]. Briefly, three replicate samples of each ash type were leached using three leachants, including two acidic leachants prepared using acetic acid (A: pH 4.93 and B: pH 2.88) and deionized water (C: pH 6.0). Leachant C was selected to simulate rain, and the pH of rain in Taiwan ranged from 5.0 to 6.0 in 2012 [32]. Following leaching with shaking at 30 rpm for 18 h, the solid/liquid ratio was 1:20. The leachates were then filtered through a 0.45-μm filter. The metal content and mutagenicity of each leachate were immediately and successively analyzed using the Ames test, as described below. The concentrations of five heavy metals (Cd, Cr, Cu, Pb, and Zn) in the leachates were determined through ICP-OES (Optima 2100DV, PerkinElmer Inc., Waltham, MA, USA). The *r*^2^ value of the calibration curve for each metal was higher than 0.995. Each leachate was analyzed in triplicate, and the heavy metal concentrations obtained were averaged.

### 2.3. Ames/Salmonella Mutagenicity Test

The Ames/Salmonella mutagenicity test was performed using a standard plate incorporation assay, with the Salmonella typhimurium strains TA98, TA100, and TA1535 with and without metabolic activation [33]. For general screening purposes, strains TA98, TA100, and TA1535 were selected based on previous studies [8,33]. Briefly, a toxic dose range experiment (cytotoxicity effect on the tester strains) was conducted first, to select an appropriate dose range for the mutagenicity test. Strains TA98, TA100, and TA1535 were each grown in nutrient broth at 37 °C for 18 h and then diluted 10^−6^- and 10^−7^-fold using phosphate-buffered saline (PBS). The collected leachates were further diluted 1-, 2.5-, 5-, and 10-fold; subsequently, 100 μL of each diluted or undiluted leachate (100, 250, or 500 μL) was mixed with diluted bacteria (100 μL) and soft agar (2 mL). Thereafter, the mixed solutions were spread on nutrient agar plates and incubated for 24 h. The cytotoxic effects of the leachates were determined by comparing the bacterial counts in the leachate and control (deionized water) groups. The leachates that were nontoxic to the Salmonella strains were subjected to a further Ames assay, as described in previous reports [8,33].

### 2.4. Statistical Analysis

Data are expressed as mean ± standard deviation (SD). The two-fold criterion and the significant effect of dose-response were selected to detect the mutagenic activity, as indicated previously [33]. The revertants in leachates are higher than the two-fold criterion of the control sets or the significant effect of dose-response of leachates can be detected, either with or without enzymatic activation for the tester, revealing that the leachates of ash display mutagenic activity. 

## 3. Results and Discussion

### 3.1. Metal Concentrations of the Leachates

Heavy metal–containing MSW may be vaporized during incineration and then condensed onto FA in the APCD. Therefore, FA generally contains higher concentrations of heavy metals than does BA [34,35]. More importantly, these heavy metals may have a long-term impact on the environment. Furthermore, the presence of metal elements, either alone or in combination, is known to be associated with the induction of genotoxicity [36,37]. For example, Ni has been reported to display mutagenic activity in the Ames assay [38]. To further evaluate the safety of these ashes, the concentrations of heavy metals in all nine leachates derived from FA and BA were determined. The five metals measured—namely Cd, Cr, Cu, Pb, and Zn—were chosen because they represent the five most abundant metal species in the various types of ash [10], and the five metals are routinely monitored by the Taiwan EPA [8]. Because Ni metal is not included within the “must test metals” by the Taiwan regulations, the concentration of Ni was not analyzed in this study. Therefore, we also determined the concentrations of the above five metal species in the BA, BFA, and PFA. In Table 1, the final pH values and metal concentrations of the BA, PFA, and BA leachates are shown. The final pH values of the various leachates increased substantially owing to the presence of alkaline substances, including SiO_2_, CaO, Al_2_O_3_, Fe_2_O_3_, and MgO [8,39]. Moreover, the concentrations of Cd and Cr in leachate B from PFA and the Cd concentration in leachate B from BFA both exceeded the limits specified in the Taiwanese regulations (Cd: 1; Pb: 5; Cr: 5; and Cu: 15 mg/L). Therefore, BFA and PFA from the selected incinerator could be classified as hazardous. In practice, in Taiwan, the levels of heavy metals within MSWI BA and FA are determined monthly by the incinerator technicians [2]. The results of these routine tests indicate that the metal concentration of FA can fluctuate and often exceeds the limits specified in the Taiwanese regulations. Hence, MSWI FA is generally recognized as hazardous.

Moreover, the concentration of Zn in leachate B from BFA, PFA, and BA was considerably higher than that of the other four metals. In addition, the metal concentration in leachate B from BFA, PFA, and BA was higher than those of leachates A and C, with the exception of Cr in BFA and BA and Pb in BA. Consistent with the previous study [5], metal concentrations were inversely proportional to the pH value of the leachants. Otherwise, as expected, the concentrations of Cu, Zn, Cd, and Pb in leachate B from BA, BFA, and PFA followed the order BFA > PFA > BA. By contrast, the concentration of Cr in leachate B from PFA was higher than that of leachate B from BFA and BA. The Cd concentration in leachate B from BFA and PFA was higher than that of leachate B from BA, because the quantity of metal leached out strongly depended on the initial metal concentration of the raw ash material and the high vaporization of Cd (Cd boiling point: 765 °C); Cd was primarily present in FA leachate [13]. As discussed, none of the TCLP leachates from BFA, PFA and BA showed mutagenic activity. Thus, none of the five tested heavy metals, nor any other substance in the nine leachates cause the leachates to be mutagenic. Furthermore, our previous study showed that several samples from leachate A from BA possess mutagenic activity, even though levels of the five heavy metals (Cd, Cr, Cu, Pb, and Zn) in TCLP leachates are below the Taiwanese regulatory limits. Nevertheless, our data support the potential reuse of both FA and BA from the current batch of MSWI. The lack of mutagenicity of leachates of FA and BA support the elimination of certain types of pretreatment before recycling, thus reducing costs.

### 3.2. Mutagenicity of the Leachates

To select an appropriate dose range for the mutagenicity test, the cellular cytotoxicity of all leachates towards tester Salmonella strains was first determined. After analyzing the toxic dose effects on tester strains, appropriate doses of leachates were further subjected to the Ames assay. In this study, the Ames test was evaluated by counting the number of revertant colonies using negative and positive controls, with spontaneous revertants being within the normal values for the three strains. Moreover, a two-fold criterion was selected to detect mutagenic activity, as indicated previously [33].

Table 2 shows the mutagenic activities of leachates A, B, and C from BFA. Here, the number of revertants in leachates A (Table 2), B (Table 3), and C (Table 4) were all below the two-fold criterion of the control sets, with or without S9 activation for strains TA98, TA100, and TA1535. Moreover, no dose-response effects of revertants were observed. Thus, these findings indicate that BFA leachates showed no detectable mutagenic activity.

Table 5, Table 6 and Table 7 show the mutagenic activities of leachates A, B, and C from PFA. Similar to the aforementioned findings, the number of revertants in leachate A (Table 5), B (Table 6), and C (Table 7) were below the two-fold criterion of the control sets, either with or without enzymatic activation for the tester, and no dose-response effects of revertants were observed, indicating that the PFA leachates showed no mutagenic activity. Therefore, both types of FA leachates, BFA and PFA, were determined to be non-mutagenic.

For comparison, the mutagenic activity of leachates A, B, and C from BA derived from the selected incinerator were also determined. As shown in Table 8, Table 9 and Table 10, the number of revertants in leachates A (Table 8), B (Table 9), and C (Table 10) were below the two-fold criterion of the control sets, with or without S9 activation, for all tester strains; moreover, no dose-response patterns of revertants were observed. These results indicate that leachates of BA showed no mutagenic activity.

Taken together, all leachates from BFA, PFA, and BA exhibited no mutagenic activity, indicating that both FA and BA may safely be reused under certain conditions. In our previous study, we determined the mutagenicity of sieved and non-sieved BA samples. In that study, although the metal contents in TCLP leachates were below the Taiwanese regulatory limits, leachate A from non-sieved and <4.75-mm-sieved BA showed mutagenicity [8]. The BA used in the previous and the current study originated from different incinerators, and the composition of FA and BA is known to differ between MSWIs [34]. This may partially explain the differing results of the two studies. 

## 4. Conclusions

Recently, two incinerators in Taiwan have attempted to recycle the FA that they produce. However, the potential environmental impacts of the FA have not yet been fully assessed. In Taiwan, the heavy metal concentrations of both FA and BA are routinely monitored and have been reported to occasionally exceed the regulatory limits. Thus, most FA is often subjected to additional pretreatment procedures to diminish the relatively high heavy metal concentrations in an effort to eliminate possible environmental and safety concerns. However, these pretreatments entail additional costs and may slow the further application of these ashes in road construction and cement manufacturing. The results of the Ames test performed in this study demonstrate that both FA and BA obtained from certain MSWIs are unlikely to be carcinogenic, indicating that reducing these pretreatments may be a prudent choice to increase efficiency without comprising safety. However, leachate A from BA did show mutagenicity in our previous report [8]. Thus, the Ames assay should be routinely applied to additional MSWI leachates. Although our results demonstrate that FA and BA obtained from the current MSWI are unlikely to be carcinogenic, other genotoxicity evaluation methods should be applied, together with the Ames assay, to confirm whether MSWI leachates have potentially toxic effects.

## Figures and Tables

**Table 1:** Metal concentrations in the TCLP leachate of MSWI ash.

Samples	Leachate Type	Final pH	Metal Concentration (mg·L^−1^)				
			Cu	Zn	Cd	Pb	Cr
Boiler Fly Ash	A (pH 4.93)	10.81	ND *	ND	0.52	0.01	0.68
	B (pH 2.88)	9.69	3.50	1310	10.8	1.01	0.70
	C (pH 6.0)	11.83	ND	ND	ND	0.00	1.18
Precipitator Fly Ash	A (pH 4.93)	9.48	ND	ND	0.32	0.04	0.93
	B (pH 2.88)	8.28	1.21	492	5.03	0.41	7.87
	C (pH 6.0)	10.01	ND	ND	0.02	0.11	0.34
Bottom Ash	A (pH 4.93)	8.82	0.23	2.61	ND	0.03	0.06
	B (pH 2.88)	7.51	0.79	94.44	0.02	0.07	0.02
	C (pH 6.0)	11.15	0.14	1.46	ND	0.41	0.02

* ND: non-detected.

**Table 2:** Revertant frequency of Salmonella tester strains TA98, TA100, and TA1535 following incubation with leachate A from BFA in presence and absence of S9 mix (mean ± SD is shown).

Number of Average Revertants (Colony Forming Unit (CFU)/Plate)						
Leachate A						
Treatment	TA98 *		TA100 *		TA1535 *	
(100 μL/plate)	−S9	+S9	−S9	+S9	−S9	+S9
Dilution 1×	37 ± 8	24 ± 3	177 ± 5	186 ± 5	16 ± 2	10 ± 3
Dilution 2.5×	35 ± 5	38 ± 1	169 ± 14	184 ± 28	16 ± 4	12 ± 6
Dilution 5×	33 ± 1	37 ± 6	178 ± 17	191 ± 8	18 ± 2	13 ± 3
Dilution 10×	39 ± 1	36 ± 5	180 ± 9	195 ± 14	18 ± 4	14 ± 5
Blank	35 ± 5	39 ± 5	198 ± 17	184 ± 12	18 ± 2	13 ± 3
Positive	1596 ± 426	392 ± 48	1316 ± 121	2086 ± 408	588 ± 73	294 ± 42

* Two-fold criterion is selected to detect the mutagenic activity. The revertants in leachates were below the two-fold criterion of the control sets, with or without S9 activation for the strains TA98, TA100, and TA1535, indicating these leachates of BFA showed no detectable mutagenic activity.

**Table 3:** Revertant frequency of Salmonella tester strains TA98, TA100, and TA1535 following incubation with leachate B from BFA in presence and absence of S9 mix (mean ± SD is shown).

Number of Average Revertants (CFU/Plate)						
Leachate B						
Treatment	TA98 *		TA100 *		TA1535 *	
(100 μL/plate)	−S9	+S9	−S9	+S9	−S9	+S9
Dilution 1×	29 ± 10	30 ± 12	170 ± 11	219 ± 51	13 ± 5	8 ± 4
Dilution 2.5×	21 ± 6	29 ± 5	175 ± 31	216 ± 44	15 ± 1	11 ± 4
Dilution 5×	24 ± 5	31 ± 7	182 ± 3	218 ± 20	20 ± 4	10 ± 3
Dilution 10×	28 ± 10	36 ± 11	184 ± 22	231 ± 12	16 ± 6	8 ± 1
Blank	35 ± 5	39 ± 5	198 ± 17	184 ± 12	18 ± 2	13 ± 3
Positive	1596 ± 426	392 ± 48	1316 ± 121	2086 ± 408	588 ± 73	294 ± 42

* See Table 2 for key.

**Table 4:** Revertant frequency of Salmonella tester strains TA98, TA100, and TA1535 following incubation with leachate C from BFA in presence and absence of S9 mix (mean ± SD is shown).

Number of Average Revertants (CFU/Plate)						
Leachate C						
Treatment	TA98 *		TA100 *		TA1535 *	
(100 μL/plate)	−S9	+S9	−S9	+S9	−S9	+S9
Dilution 2.5×	32 ± 4	30 ± 3	166 ± 25	202 ± 34	15 ± 1	11 ± 1
Dilution 5×	28 ± 7	30 ± 5	174 ± 7	192 ± 45	11 ± 3	10 ± 5
Dilution 10×	27 ± 8	35 ± 6	188 ± 10	209 ± 11	19 ± 2	12 ± 3
Blank	35 ± 5	39 ± 5	198 ± 17	184 ± 12	18 ± 2	13 ±3
Positive	1596 ± 426	392 ± 48	1316 ± 121	2086 ± 408	588 ± 73	294 ± 42

* See Table 2 for key.

**Table 5:** Revertant frequency of Salmonella tester strains TA98, TA100, and TA1535 following incubation with leachate A from PFA in presence and absence of S9 mix (mean ± SD is shown).

Number of Average Revertants (CFU/Plate)						
Leachate A						
Treatment	TA98 *		TA100 *		TA1535 *	
(100 μL/plate)	−S9	+S9	−S9	+S9	−S9	+S9
Dilution 1×	34 ± 8	36 ± 2	162 ± 8	157 ± 7	15 ± 2	15 ± 6
Dilution 2.5×	38 ± 9	37 ± 4	165 ± 22	175 ± 23	15 ± 2	13 ± 7
Dilution 5×	41 ± 6	37 ± 3	164 ± 14	176 ± 4	18 ± 0	10 ± 3
Dilution 10×	32 ± 8	41 ± 3	171 ± 15	184 ± 12	18 ± 2	15 ± 4
Blank	35 ± 5	38 ± 5	198 ± 17	183 ± 10	18 ± 2	13 ± 3
Positive	1596 ± 426	518 ± 159	1316 ± 121	2170 ± 461	588 ± 73	294 ± 42

* Two-fold criterion is selected to detect the mutagenic activity. The revertants in leachates were below the two-fold criterion of the control sets, with or without S9 activation for the strains TA98, TA100, and TA1535, indicating these leachates of PFA showed no detectable mutagenic activity.

**Table 6:** Revertant frequency of Salmonella tester strains TA98, TA100, and TA1535 following incubation with leachate B from PFA in presence and absence of S9 mix (mean ± SD is shown).

Number of Average Revertants (CFU/Plate)						
Leachate B						
Treatment	TA98 *		TA100 *		TA1535 *	
(100 μL/plate)	−S9	+S9	−S9	+S9	−S9	+S9
Dilution 1×	34 ± 3	33 ± 4	171 ± 35	176 ± 12	11 ± 2	13 ± 5
Dilution 2.5×	46 ± 1	29 ± 7	153 ± 6	173 ± 19	14 ± 3	11 ± 1
Dilution 5×	41 ± 9	43 ± 7	158 ± 12	182 ± 16	16 ± 4	8 ± 4
Dilution 10×	31 ± 9	39 ± 1	193 ± 7	175 ± 10	14 ± 2	11 ± 2
Blank	35 ± 5	39 ± 5	198 ± 17	183 ± 10	18 ± 2	13 ± 3
Positive	1596 ± 426	392 ± 48	1316 ± 121	2170 ± 461	588 ± 73	294 ± 42

* See Table 5 for key.

**Table 7:** Revertant frequency of Salmonella tester strains TA98, TA100, and TA1535 following incubation with leachate C from PFA in presence and absence of S9 mix (mean ± SD is shown).

Number of Average Revertants (CFU/Plate)						
Leachate C						
Treatment	TA98 *		TA100 *		TA1535 *	
(100 μL/plate)	−S9	+S9	−S9	+S9	−S9	+S9
Dilution 1×	23 ± 1	28 ± 3	168 ± 20	195 ± 35	13 ± 2	13 ± 3
Dilution 2.5×	33 ± 8	30 ± 9	187 ± 17	163 ± 21	14 ± 6	14 ± 4
Dilution 5×	28 ± 3	39 ± 4	179 ± 10	180 ± 13	14 ± 3	14 ± 8
Dilution 10×	37 ± 11	38 ± 5	195 ± 19	172 ± 15	14 ± 1	15 ± 3
Blank	35 ± 5	39 ± 5	198 ± 17	183 ± 10	18 ± 2	13 ± 3
Positive	1596 ± 426	392 ± 48	1316 ± 121	2170 ± 461	588 ± 73	294 ± 42

* See Table 5 for key.

**Table 8:** Revertant frequency of Salmonella tester strains TA98, TA100, and TA1535 following incubation with leachate A from BA in presence and absence of S9 mix (mean ± SD is shown).

Number of Average Revertants (CFU/Plate)						
Leachate A						
Treatment	TA98 *		TA100 *		TA1535 *	
(100 μL/plate)	−S9	+S9	−S9	+S9	−S9	+S9
Dilution 1×	17 ± 4	36 ± 8	203 ± 27	208 ± 11	11 ± 4	16 ± 2
Dilution 2.5×	23 ± 8	40 ± 10	178 ± 14	200 ± 29	11 ± 3	13 ± 3
Dilution 5×	20 ± 5	28 ± 4	202 ± 9	201 ± 24	14 ± 5	13 ± 3
Dilution 10×	20 ± 1	27 ± 6	170 ± 6	202 ± 11	14 ± 6	11 ± 3
Blank	35 ± 5	39 ± 5	178 ± 15	184 ± 12	14 ± 3	13 ± 3
Positive	1596 ± 426	392 ± 48	2072 ± 246	2086 ± 408	560 ± 106	294 ± 42

* Two-fold criterion is selected to detect the mutagenic activity. The revertants in leachates were below the two-fold criterion of the control sets, with or without S9 activation for the strains TA98, TA100, and TA1535, indicating these leachates of BA showed no detectable mutagenic activity.

**Table 9:** Revertant frequency of Salmonella tester strains TA98, TA100, and TA1535 following incubation with leachate B from BA in presence and absence of S9 mix (mean ± SD is shown).

Number of Average Revertants (CFU/Plate)						
Leachate B						
Treatment	TA98 *		TA100 *		TA1535 *	
(100 μL/plate)	−S9	+S9	−S9	+S9	−S9	+S9
Dilution 1×	14 ± 4	37 ± 10	211 ±33	196 ± 6	11 ± 4	14 ± 3
Dilution 2.5×	14 ± 3	41 ± 3	188 ±13	190 ± 8	14 ± 2	15 ± 6
Dilution 5×	15 ± 3	42 ± 7	188 ±26	213 ± 16	13 ± 1	16 ± 3
Dilution 10×	15 ± 3	30 ± 12	190 ±34	188 ± 44	11 ± 2	12 ± 7
Blank	13 ± 5	39 ± 5	178 ±15	184 ± 12	14 ± 3	13 ± 3
Positive	253 ± 89	392 ± 48	2072 ±246	2086 ± 408	560 ± 106	294 ± 42

* See Table 8 for key.

**Table 10:** Revertant frequency of Salmonella tester strains TA98, TA100, and TA1535 following incubation with leachate C from BA in presence and absence of S9 mix (mean ± SD is shown).

Number of Average Revertants (CFU/Plate)						
Leachate C						
Treatment	TA98 *		TA100 *		TA1535 *	
(100 μL/plate)	−S9	+S9	−S9	+S9	−S9	+S9
Dilution 2.5×	31 ± 5	27 ± 7	181 ± 17	216 ± 32	9 ± 1	11 ± 2
Dilution 5×	24 ± 3	27 ± 4	199 ± 14	199 ± 24	12 ± 3	10 ± 2
Dilution 10×	31 ± 7	25 ± 8	206 ± 13	205 ± 10	12 ± 2	14 ± 3
Blank	35 ± 5	39 ± 5	178 ± 15	184 ± 12	14 ± 3	13 ± 3
Positive	1596 ± 426	392 ± 48	2072 ± 246	2086 ± 408	560 ± 106	294 ± 42

* See Table 8 for key.
